# Effects of Virtual Speaker Density and Room Reverberation on Spatiotemporal Thresholds of Audio-Visual Motion Coherence

**DOI:** 10.1371/journal.pone.0108437

**Published:** 2014-09-30

**Authors:** Narayan Sankaran, Johahn Leung, Simon Carlile

**Affiliations:** Auditory Neuroscience Laboratory, School of Medical Sciences, The University of Sydney, Sydney, NSW, Australia; University of Melbourne, Australia

## Abstract

The present study examined the effects of spatial sound-source density and reverberation on the spatiotemporal window for audio-visual motion coherence. Three different acoustic stimuli were generated in Virtual Auditory Space: two acoustically “dry” stimuli via the measurement of anechoic head-related impulse responses recorded at either 1° or 5° spatial intervals (Experiment 1), and a reverberant stimulus rendered from binaural room impulse responses recorded at 5° intervals *in situ* in order to capture reverberant acoustics in addition to head-related cues (Experiment 2). A moving visual stimulus with invariant localization cues was generated by sequentially activating LED's along the same radial path as the virtual auditory motion. Stimuli were presented at 25°/s, 50°/s and 100°/s with a random spatial offset between audition and vision. In a 2AFC task, subjects made a judgment of the leading modality (auditory or visual). No significant differences were observed in the spatial threshold based on the point of subjective equivalence (PSE) or the slope of psychometric functions (β) across all three acoustic conditions. Additionally, both the PSE and β did not significantly differ across velocity, suggesting a fixed spatial window of audio-visual separation. Findings suggest that there was no loss in spatial information accompanying the reduction in spatial cues and reverberation levels tested, and establish a perceptual measure for assessing the veracity of motion generated from discrete locations and in echoic environments.

## Introduction

Various experiments have sought to determine the nature of the spatiotemporal integration window for audio-visual motion [1]–[3]. To probe this question, studies typically deliver moving auditory stimuli using an array of sequentially activated speakers in free-field [4]–[7], or over headphones by measuring Head Related Impulse Responses (HRIRs) and rendering a *Virtual Auditory Space (VAS)* [8]. Irrespective of the delivery method, there are a number of unresolved issues in the process.

In the generation of acoustical motion, moving the sound source itself mechanically has the advantage of real-world coherence [9], [10]. However, physical constraints such as background motor noise, restricted speeds and limited spatial extents present numerous disadvantages experimentally. Instead, the percept of motion is usually created by sequentially activating discrete stationary sound-sources. Whether these are physical speakers placed in free field arrays or stimuli rendered in VAS via the measurement of HRIRs (see methods), the changes in acoustical cues are quantized, resulting in a loss of spatial information. While the resulting moving stimulus may be perceived as spatially continuous, other psychophysical consequences of this reduction in cue density remain unclear. This is an important consideration given that a clear understanding of the mechanisms underlying auditory motion perception remain outstanding. Typical step-sizes utilized in auditory motion studies range from approximately 2° to 6° [4], [6], [11]. Intuitively, a perceptual limit of this quantization can be estimated from the minimum audible movement angle (MAMA), defined as the minimum spatial extent required for a sound to elicit a motion percept [12]. However, reported values differ depending on velocity and spectral content, confounding a systematic description of MAMA across any one parameter. Using moving stimuli generated by stereo balancing a 500 Hz tone across two speakers, Grantham [13] reported MAMAs ranging from 5° to 21° at source velocities of 15°/s and 90°/s respectively. Perrot and Marlborough [10] found MAMAs ranging from 0.9° to 1.6° using a speaker that rotated at 20°/s with a 500 Hz–8 kHz pink noise stimulus. The small but statistically significant difference depended on whether onset and offset cues were provided to the listeners. In contrast, Chandler and Grantham [9] reported a value of 5.6° using 500–10 kHz “wideband” noise delivered by a speaker moving at 20°/s, increasing to 14.4° at a velocity of 90°/s. Taken together, these studies describe a metric that is highly variable, with the only commonality being the increase with velocity. Further complicating the issue, as suggested in Grantham [13] and confirmed in Carlile and Best [14] and Freeman et al. [15] (2014), velocity per se is not a salient cue in auditory motion perception. Given the increasing number of auditory motion studies that use a discrete-sequential presentation technique, a goal of the present study is to compare auditory motion perception of the finest spatial discretization (1°) against one that is commonly used (5°) using wide-band stimuli at various velocities.

Experiments often present a moving auditory stimulus that is anechoic. However, everyday environments contain reverberant energy due to sound-reflecting surfaces. Despite its ubiquity, little is known about the perceptual effects of reverberation outside its influence on stationary sound sources [16]–[19]. Such studies have demonstrated that, though the ratio of direct to reverberant energy (D/R) provides a direct cue to source depth that would be unavailable to the listener under anechoic conditions [20], [21], the interference of direct and reflected sound at the listener's ears can decorrelate the binaural cues, thereby diminishing localization ability [16]. One goal of the current study is to explore the nature of this trade-off when a source is in motion. Rather than utilizing a very echoic environment, where reflected sound obviously diminishes localizability, the current study examines reverberation levels found in typical listening rooms. In doing so, the perceptual impact of reverberation in the most common listening environments can be better understood.

A body of neurophysiological and psychophysical evidence suggests that specific motion-detectors are present at early stages of visual processing [22]. In contrast, there is no similar evidence of similar low level encoding in the auditory periphery [23]. However, various models of auditory motion have been proposed. One such model that is widely quoted is the “snapshot” hypothesis, whereby motion is perceived via the sequential localization and comparison of a number of static snapshots [24]. In this context, reverberation then may also degrade the acuity of motion perception since movement is inferred from the same static cues that reverberation degrades. Consistent with this notion, in a motion detection task, Saberi and Petrosyan [25] reported a rapid deterioration in performance from supra-threshold to chance level as the amount of correlation in the binaural acoustical cues decreased.

Traditionally, investigations into these issues are limited to unimodal approaches. Here, we present audio-visual motion in order to explore the effects of acoustic spatial quantization and reverberation on the spatiotemporal integration window. Models of optimal integration suggest that overall localization uncertainty is minimized via the optimal weighing of each sensory input based on the reliability of their constituent cues [26]–[28]. Such models account not only for ventriloquism, where visual cues dominate perception, but describe a two-way interaction in which auditory and visual streams concurrently influence each other [29], [30]. Other studies have shown this holds for moving audio-visual sources [5], [31]–[33]. In the current study, subjects compared the relative times at which moving virtual auditory and visual targets were perceived to pass the midline.

In Experiment 1, auditory motion was spatially constrained to two step-sizes: a densely sampled 1° and the sparser 5° quantization. This reduction in cue density necessitates a spread of acoustical information from a 1° to a 5° window, which may elicit greater spatial uncertainty. In Experiment 2, relevant room acoustical information was included in the construction of the VAS, using binaural room impulse responses (BRIRs) recorded *in situ* in 5° step-sizes. If the reverberation perceptibly decorrelated the binaural cues, we expected the spatial uncertainty of the reverberant stimuli to be even greater than that of the anechoic stimuli. Throughout this study, the visual stimuli remained unchanged (see methods), ensuring invariant visual localization cues across all auditory conditions. Given this, and the significantly greater spatial resolution of the visual system, the visual stimulus served as a reference, allowing for an unambiguous comparison between acoustic conditions. We thus hypothesized that the reduction in cue density and reverberation would increase task difficulty, making the judgment about which modality was leading harder. This would be reflected by an increase in the spread of the distribution, resulting in greater variance of a fitted Gaussian function (β). The point of subjective audio-visual equality (PSE) was also measured for the three acoustic conditions, though the effects of auditory uncertainty on this parameter are harder to predict. It is important to note that even though vision has a significantly greater spatial resolution, the current study provides insight into the effects of quantization and reverberation through the relative comparisons across acoustic conditions.

## Experiment 1: Quantization of Auditory Space

### Methods

#### Participants

Six subjects (five male, one female) participated in the experiment. All subjects had normal hearing as confirmed by audiometric screening.

#### Ethics Statement

Written informed consent was provided and experiments were approved by the Human Research Ethics Committee of the University of Sydney (HREC number 15278).

#### Stimuli

The recording procedure and rendering of motion in VAS is briefly outlined below. For a more detailed description see Carlile [8]. Individualized blocked ear HRIRs [14], [34] were measured under anechoic conditions by securing microphones in the ear canals using medical grade silicon gel (Polyvinylsiloxane). The subjects' head was stabilized by a chin-rest and monitored using a head-tracker (InterSense IC3). One-second exponential sine sweep stimuli [35] were presented by a speaker (Audience A3) mounted at the apex of a robotic arm that moved along a radial arc 1 meter from the listener. Measurements were taken from −90° to +90° along the audio-visual horizon in 1° increments.

**Figure 1:**
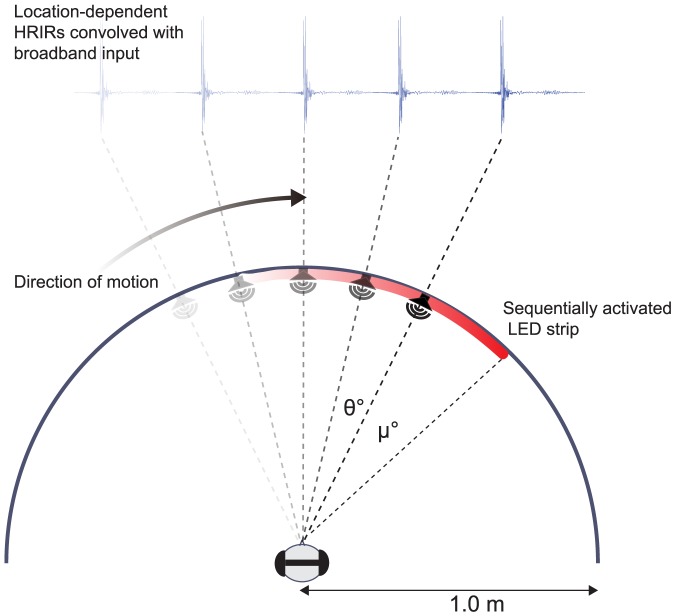
Motion generation and experimental setup. Subjects were positioned at the centre of a 1 meter radial arc extending from −90° to 90° along the audio-visual horizon. Their heads remained in a fixed position, stabilized by a chin rest and motion direction was alternated between trials. HRIRs from adjacent positions spaced by θ° were convolved with the broadband input stimulus before being sequentially played to achieve apparent auditory motion, presented via headphones. The auditory stimuli were spatially offset from visual stimuli by an amount μ° throughout the motion path. Subjects made a 2AFC judgement of the leading modality as it crossed the approximate midline.

The responses of the recording microphone and stimulus speaker were then deconvolved from the HRIRs. Figure 1 summarises the process by which moving auditory stimuli were generated. First, a broadband white noise (300 Hz to 16 kHz) of the total trial duration was generated. This was then filtered with a series of bandpass filters (from 400 to 16 kHz, equally spaced at 200 Hz with a bandwidth of 100 Hz) and amplitude modulated at 20 Hz. Such a stimulus provided a high level of modulation coherence so as to encourage perceptual object formation [36]. Finally, each segment of the noise stimulus was convolved with left and right HRIRs corresponding to each recording position (1° or 5° steps), the duration of each segment being determined by the chosen velocity of motion (see below). Subjects indicated (via qualitative feedback) that the auditory stimuli were externalized and easily localizable, which is consistent with our previous findings using similar stimuli [37]. Apparent motion was created by sequentially playing the convolved output corresponding to adjacent HRIR positions along the radial trajectory. Different velocities were generated by changing the duration per segment of noise at each quantized step; e.g. a 100°/s stimulus will have a 10 ms duration time per 1°. The 5° quantized stimulus followed the same procedure, however HRIR positions were constrained to 5° increments and the duration per position was correspondingly increased (i.e. a 100°/s stimulus would have a duration of 50 ms per 5° step). In addition, the final and initial conditions of the convolved signal from adjacent filters were combined in software (MATLAB 8.0, The MathWorks Inc) to ensure a smooth continuous signal. The rendered auditory signal was delivered to a pair of Beyer-Dynamic DT990 open-back headphones via an RME Fireface 400 audio interface, using the Psychophysics Toolbox extensions [38]–[40] to ensure sample-accurate playback timing. All recording and digital processing was performed at a 48 kHz-sampling rate.

To generate the visual stimulus, an array of high-density LEDs spaced by 1.8° was arranged in a strip along the same radial path as the rendered auditory stimuli. For each individual LED, the brightness, colour, and activation timing were controlled using a WS2801 integrated controller with microsecond accuracy. An Arduino Mega2560 USB platform connected to a Matlab interface was used to power and control the LED strip. Apparent visual motion was produced by the sequential ‘on-off’ activation of adjacent LEDs along the strip, again, specifying velocity as a function of time per pulse. All subjects reported that apparent visual motion was smooth for all velocities examined, with the stimulus eliciting the percept of a moving line along the radial path.

Playback timing between the auditory and visual stimuli was calibrated by measuring the excitation of two photodiodes placed at various locations along the LED strip while simultaneously recording audio output. In doing so, systematic latencies in LED activation were adjusted to ensure temporal onset accuracy of auditory and visual stimuli (see below).

#### Procedure

Trials consisted of moving auditory and visual stimuli presented along a common radial trajectory with a 1 m radius along the frontal audio-visual horizon. The trajectory subtended 140° around the subject whose head was aligned using reference lasers and stabilized by a chinrest (figure 1). The two modalities were temporally aligned, but spatial congruency was varied such that audition with respect to vision was either leading or lagging in the direction of motion. This was done by presenting the visual stimulus along a constant trajectory from −70° to +70° and varying the auditory start and end points to achieve the desired spatial offset. To avoid motion after-effects, the stimuli direction (leftward or rightward) alternated on a trial-to-trial basis. In a 2AFC task, observers were asked to track the visual stimulus with their eyes and indicate the perceived leading modality as the stimuli crossed the approximate midline, registering their response on a keyboard. For each quantization level (1° vs. 5°), auditory and visual stimuli were presented at three velocities; 25°/s, 50°/s and 100°/s, resulting in total stimulus durations of 5.8, 2.9 and 1.45 seconds respectively. Auditory and visual stimuli were spatially offset by randomly varying the starting location of the auditory stimuli to one of nine possible values (Table 1). Here, positive offsets indicate a visual lead; negative offsets indicate an auditory lead and zero represents spatiotemporal equality. A testing block consisted of 90 trials (10 repeats per displacement) at a given velocity and for a given acoustical condition. Psychometric functions (PF) were fitted to the results and analyzed as described below. Subject responses were fitted to a cumulative Gaussian distribution using a maximum likelihood estimation function. The lapse rate of the PF fit was maximally limited to 0.06 to account for errors due to stimulus-independent effects [41], [42].

**Table 1:** Audio-Visual Spatial displacements.

Velocity (°/sec)	μ (°)
25	0, ±1.25, ±2.5, ±5, ±10
50	0, ±2.5, ±5, ±7.5, ±10
100	0, ±5, ±10, ±15, ±20

At each velocity, congruence between auditory and visual stimuli was offset by one of nine randomized values. Positive offsets indicate an auditory lag; negative values indicate an auditory lead. Zero represents spatiotemporal equality.

From each PF, two values were extracted. Firstly, the Point of Subjective Equality (PSE), here defined as the domain value at the inflection of the cumulative PF. Secondly, the Slope (β), defined as the variance of the Gaussian fit. PFs were parametrically bootstrapped based on a maximum likelihood model [43] (n = 1000) in order to obtain 95% confidence limits solely for comparing within-subject data. All relevant experimental data is available at http://dx.doi.org/10.6084/m9.figshare.978755 including individual subject data.

### Results

**Figure 2:**
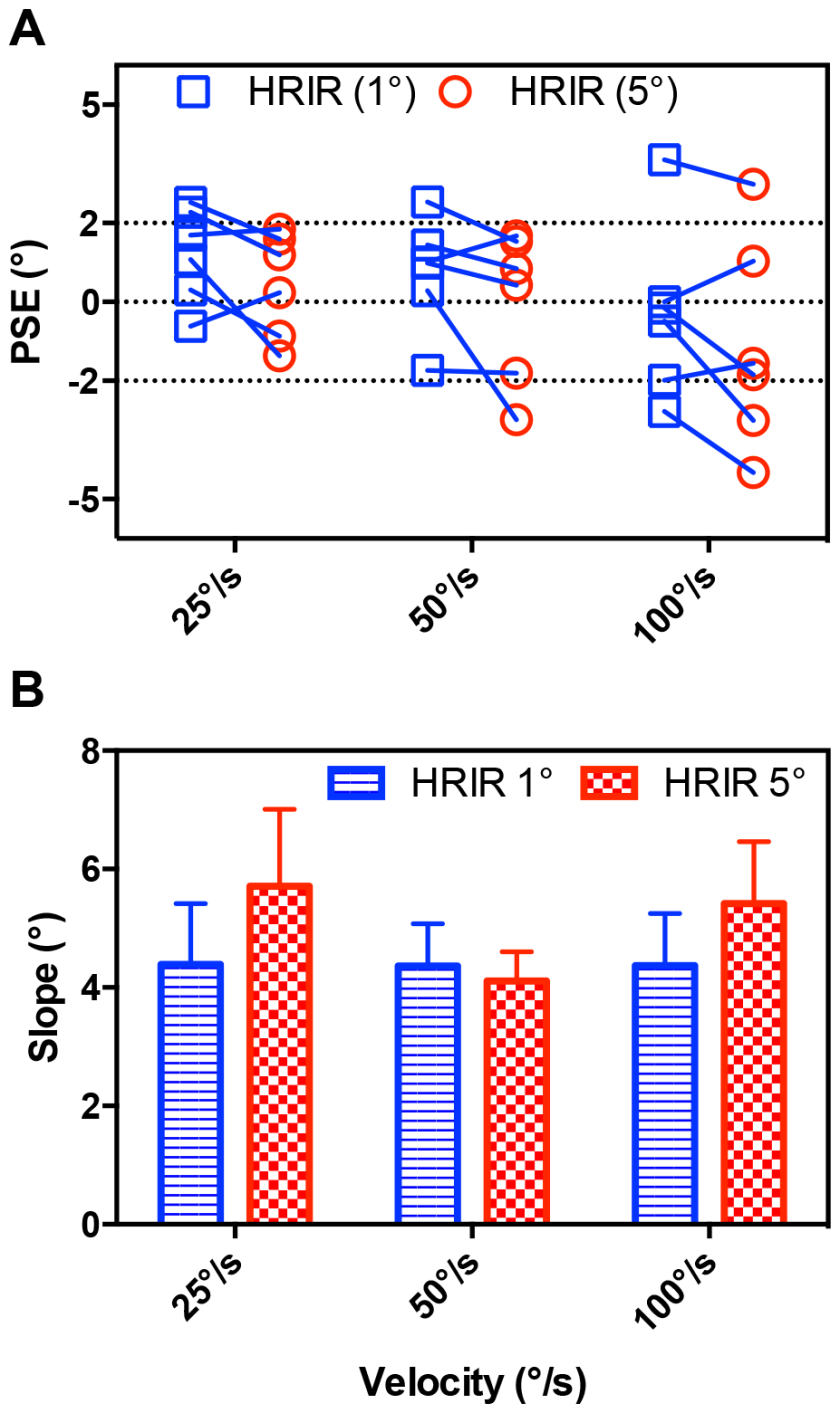
Results for Experiment 1. (A) Individual PSEs from the two acoustic conditions for all six subjects. Blue squares and red circles denote values from HRIR (1°) and HRIR (5°) respectively. (B) Group mean βs shown in blue and red for HRIR (1°) and HRIR (5°) respectively at the three experimental velocities. Error bars indicate between-participants standard errors.

PSEs for experiment 1 are shown in Figure 2A for all subjects (see also Table S1). Positive PSEs equate to a physically leading visual stimulus, indicating a perceptual tendency to judge the auditory stimulus as leading when both stimuli had spatiotemporal equality. We refer to this as an auditory lead bias. Similarly, negative PSEs denote a visual lead bias. The results show substantial across-subject variability for a given acoustic condition and velocity. While PSEs were slightly greater in the HRIR 1° condition, this general trend did not reach statistical significance. A repeated-measures ANOVA was performed to examine the effects of both quantization level (HRIR 1° vs. HRIR 5°) and stimulus velocities (25 vs. 50 vs. 100°/s). No significant main effects were observed for quantization level (F = 5.74, p = 0.12) or velocity (F = 2.33, p = 0.15). The interaction between quantization levels and velocity was also insignificant (F = 0.005, p = 0.995).

The group means of the psychometric function slopes (β) are plotted in Figure 2B. Though there was a general trend of higher βs in the HRIR 5° condition, a repeated measures ANOVA showed that the effects of quantization level on β was not statistically significant (*F = 5.4, p = 0.07*). Interestingly, β was statistically equivalent across the three velocity conditions (*F = 0.870, p = 0.45*). Furthermore, the interaction between quantization level and velocity was also statistically insignificant (F = 1.05, p = 0.39).

## Experiment 2: Reverberant Auditory Motion

### Methods

In order to examine the effects of reverberation, Binaural Room Impulse Responses (BRIRs) were measured *in situ* i.e. in the experimental testing room (17 m^3^, RT_60_ ∼200 ms), ensuring that a veridical amount of room acoustical information was included in the recordings. BRIR recordings were made as in Experiment 1, with the exception that 5-second exponential sine sweeps were used as the impulse response recording stimuli. These were presented over a Fostex PMO.4n dual-cone speaker that was positioned manually in 5° increments. This longer recording stimulus was necessary to ensure that the relevant reverberant acoustics were properly characterized (see below). The duration of the test stimulus was determined as per the method and velocities of Experiment 1.

**Figure 3:**
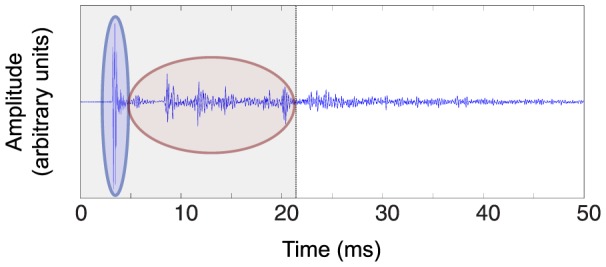
Example Binaural Room Impulse Response (BRIR). BRIR measured using microphones positioned inside the ear canal of one subject. Pilot testing determined that the shaded area contained perceptually relevant information while the subsequent reverberant tail (>21 ms) was discarded. The regions inside the blue and red ellipses represent direct and reverberant energy respectively. Six early-reflected peaks are visible in the preserved BRIR.

Major reflective peaks were found in the first 21 ms of all BRIRs measured (Figure 3), which was preserved and convolved with the input stimulus. Pilot testing confirmed that there was no perceptual difference between stimuli rendered from the entire BRIR versus one which only used the first 21 ms of the filter (i.e. the reverberant tail contained no perceptually significant detail). Further testing and estimation of D/R also verified that the reverberant stimuli contained a salient amount of room information (see Discussion). Motion was then generated as described earlier (see Figure 1) and the experimental procedure followed that of Experiment 1.

### Results

**Figure 4:**
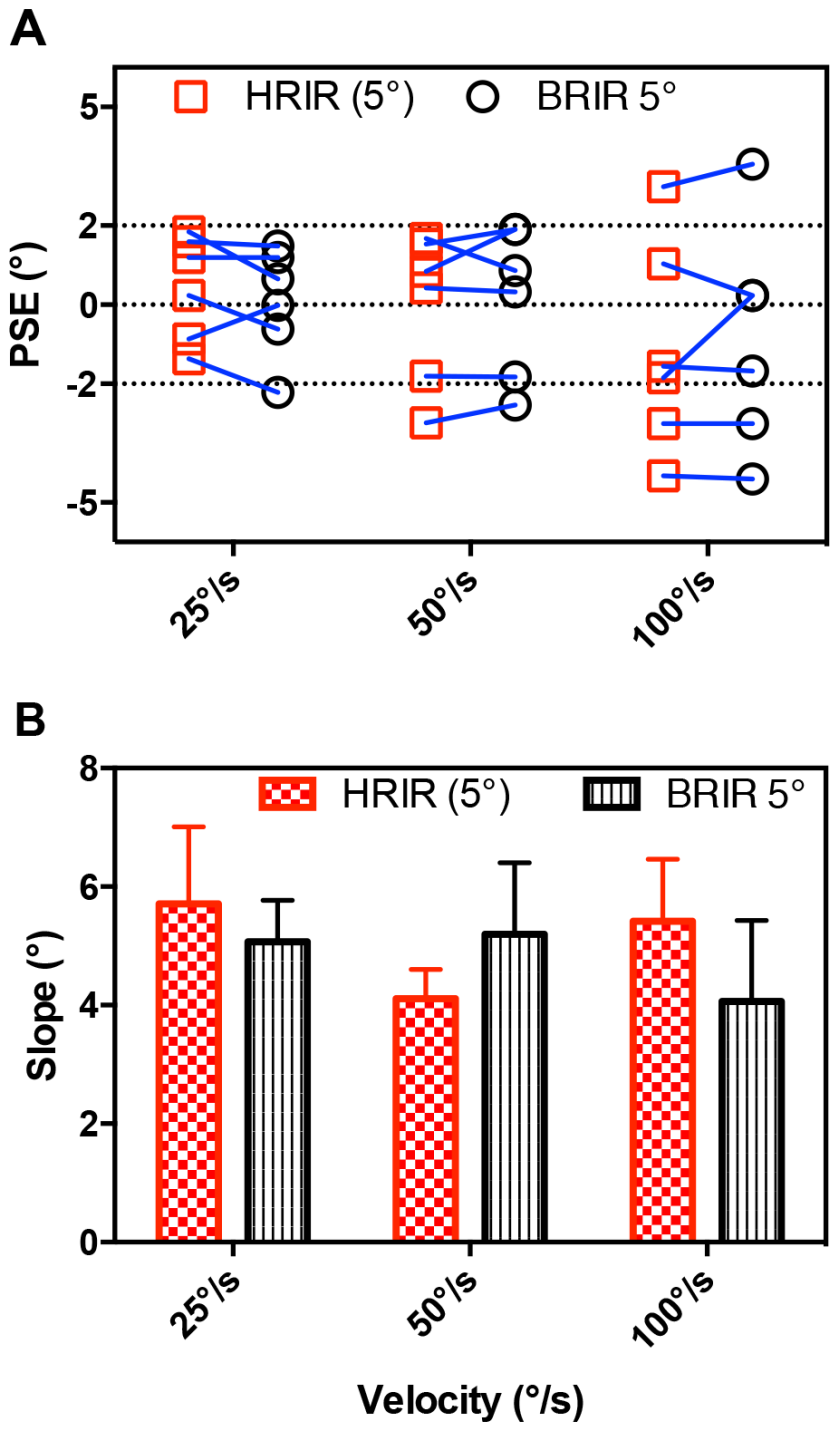
Results for Experiment 2. (A) Individual PSEs from the reverberant condition (BRIRs; black circles) presented alongside the anechoic data experiment 1 (HRIRs; red squares) for all six subjects. Both auditory spaces are spatially quantized at 5° step-sizes. (B) Group mean βs. Error bars indicate between-participants standard errors.

PSEs for experiment 2 are shown in Figure 4A (BRIR 5°), plotted alongside PSEs corresponding to the anechoic condition of equal spatial sampling from experiment 1 (HRIR 5°) for comparison. A 2×3 repeated measures ANOVA was performed and no statistically significant effects on PSEs were observed for acoustical condition (F = 0.10, p = 0.77) or velocity (F = 1.57, p = 0.26). The interaction between velocity and acoustical condition was also not significant (F = 0.75, p = 0.50).

Group mean βs from reverberant conditions are shown in Figure 4B (BRIR 5°), presented alongside the results from experiment 1 (HRIR 5°). Contrary to our expectations, no significant differences were observed between reverberant and anechoic conditions (2×3 repeated measures ANOVA; F = 0.11, p = 0.75) or stimulus velocities (2×3 repeated measures ANOVA; F = 0.49, p = 0.63) and the interaction was insignificant (F = 1.25, p = 0.33).

### Discussion

Visual cues remained constant throughout both experiments. Given this, our results suggest that the reduction in spatial cue density and the interaural decorrelation imparted by reverberation cause no perceptually significant loss of auditory spatial information, at least across the range of parameter space tested. Additionally, β was invariant across velocity in both experiments, suggesting that the threshold for perceptible audio-visual separation had a constant spatial arc. We discuss these findings in the context of several stimulus and task-related factors.

#### Multimodal Interaction

While this study compared between different acoustical conditions, we took advantage of the perceptual separability between the visual and auditory stimuli to use a visual stimulus as the spatiotemporal reference, thus mitigating any potential acoustical confounds. Stimuli in both modalities were distinct and highly localizable and subjects reported no multisensory integration or fused percept. Prior knowledge of the independent nature of the modalities may also have contributed in preventing any sensory integration. While there is evidence suggesting that cross-modal interactions may occur despite the lack of such integration [4], this has only been shown in a split attention task with short, narrow band stimuli. In this study we provided subjects with access to the full range of auditory localisation cues and emphasised the need to attend to both modalities.

#### Velocity Invariant Spatial Window

The slope of the PF (β) reflects the level of uncertainty in the psychophysical judgement rather than accuracy [41], [42]. In this context it reflects the objective difficulty in resolving the location of auditory from visual stimuli. Given this, β enables inferences about the magnitude of the spatiotemporal window of audio-visual separation. Interestingly, results from Experiments 1 and 2 found no significant difference in β across experimental velocities. Given that audio-visual offsets (Table 1) were defined spatially and β values were thus calculated from a PF in the spatial domain, these data suggest that the spatial resolution of the audio-visual system is constant across the parameter space tested in the current study. Because stimulus velocity was constant, this corresponds to a finer temporal window of audio-visual separation for higher velocities. Such a decrease in temporal variability with increasing stimulus velocity is consistent with a previous motion extrapolation study [11] in which subjects registered the arrival times of a moving auditory stimulus crossing a stationary visual fixation. Though not the focus of their study, they found a decrease in the variability of estimated arrival times as stimulus speed increased (from a 160 ms standard deviation at 17°/s to 100 ms at 47°/s, read from their Figures 2 and 3). Furthermore, the lack of significant differences in β across 1° and 5° VAS suggests that the invariance in β between anechoic and reverberant conditions, where both stimuli were quantized at 5°, represents a genuine perceptual threshold, whereby a judgment of the leading modality in the latter condition (echoic vs. anechoic conditions) may have been constrained by the resolving capability of the auditory system rather than a physical limit imposed by the spatial quantization of the stimulus.

#### MAMA

The perceptual relevance of a reduction in the density of acoustic cues can be linked to measures of the MAMA. Findings from prior studies have reported MAMA's ranging from 1° to 21°, depending on stimulus velocity and bandwidth [9], [13]. PSEs in the current study had a magnitude of less than 5°, ranging from −4.41° to 3.6° (Figures 2,4 and Table S1), and βs did not significantly differ across 1° and 5° VAS (Figure 2). Together, these results suggest that the 5° quantization of auditory space is still sub-threshold, thus resulting in no perceptually significant discretization of auditory motion for the broadband stimuli and velocities tested. Consistent with this, subjective feedback from pilot tests confirmed that motion was perceptually smooth at 5° step-sizes (see also Feinkohl et al. [37]) suggesting that the sparser auditory sampling resulted in no loss of spatial resolution.

#### Reverberation Level and Room Characteristics

As discussed previously, a clear body of evidence demonstrates that reverberation degrades the quality of acoustic cues utilized for static localization [16]. Consequently, we predicted that reverberation would degrade auditory motion perception and thus alter the spatiotemporal dynamics between vision and audition. In light of this, the lack of significant difference in both the PSE and β between anechoic and reverberant conditions was surprising.

The present study sought to examine reverberation in everyday listening rooms, with less reflected energy then that used in Hartmann [16]. The perceptual quality of reverberation in the BRIR recording (and testing) room (RT_60_ ∼200 ms) is therefore of interest. Though the reverberation level of the environment was relatively lower than previous studies, qualitative listening confirmed that the reverberation was perceptible (particularly so in contrast to the anechoic chamber environment); the stimulus had a vastly different sound quality, contained more “presence” and yielded a more externalized percept than the anechoic stimulus. To obtain quantitative evidence of this perceptual difference, we consider the difference in D/R between the two environments. Using techniques outlined by Jeub et al. [44], the D/R of the anechoic and reverberant impulses were estimated to be 20.3 dB and 3.9 dB respectively. Zahorik [45] determined the JND for D/R sensitivity in VAS to be 6 dB, which is substantially lower than the 16.4 dB difference between acoustical conditions found in the present study. This strongly suggests that the BRIRs obtained in our testing room contained a perceptually salient level of reverberation.

Even though reverberation levels in our experiment were above perceptual threshold, geometric properties of the room may be such that the *precedence effect* remediated the deleterious effects of reverberation on localization [16], [46]. The precedence effect refers to the perceptual ability to suppress late-arriving signals in order to extract localization cues in the onset waveform. The mechanism by which precedence operates varies depending on the temporal separation of subsequent signals [47]. When the temporal spacing of direct and reflected signals are proximate (0–1 ms interval), a fused image is observed rather than two separate sounds, and the perceived direction is a complex average of the two waveforms, referred to as *localization summation* [48]. Note however that the direct and first-reflected peaks of BRIRs in the current study are separated by approximately 2.5 ms (Figure 3). For intervals of this magnitude, direct and reflected waveforms maintain a fused percept but the perceived direction is dominated by the initial signal. In such cases of *localization dominance* [47], reverberation still holds perceptual weighting, conveying qualitative information about the environment, but directional information is extracted solely from the direct waveform. Even beyond the echo threshold, when fusion ceases and two separate images are heard, *discrimination suppression* caused by the presence of the direct signal can inhibit processing of the reflected signal's spatial cues. The echo threshold varies according to several acoustic properties of the surrounds, but widely reported values lie between 3 and 10 ms [47]. Thus, with a temporal delay of 2.5 ms between direct and reflected signals, subjects presented with reverberant stimuli may have recovered direct onset cues due to the combined processes of localization dominance and discrimination suppression. Such a process of echo suppression would result in reverberant stimuli with directional cues akin to anechoic stimuli, accounting for the result of the present study. Supporting this, the environment in which Hartmann [16] showed the disruptive effect of reverberation on static localization was highly echoic, with an RT60 of 4 seconds. Though the precedence effect operates at time periods proximal to onset, research suggests that precedence has a longer time-course for ongoing sounds due to multiple onsets brought about by local energy fluctuations [49], [50]. Given that our stimuli consist of a concatenation of multiple discreet signals, it is possible that auditory localization may still have been influenced by mechanisms relating to precedence. An interesting question for future consideration is whether a reverberant source in motion alters the thresholds of fusion, dominance and suppression or gives rise to entirely new perceptual phenomena.

### Concluding Remarks

The current study explored the effects of spatial quantization and reverberation on auditory motion perception. In order to do this, three different acoustic stimuli were rendered in VAS: two anechoic stimuli which differed in their spatial cue density, and a reverberant stimulus recorded *in situ* in order to capture veridical room acoustics. These stimuli were presented with a temporally synchronous but spatially varied co-moving visual stimulus with constant cues, thereby serving as a localization reference. No significant differences were found in the PSE or β between conditions in which the auditory spatial sampling was discretised to 1° and 5° or between conditions in which the auditory stimuli was anechoic and reverberant, suggesting that listeners lacked sensitivity to the quantization and reverberation levels tested in the current study. The MAMA and precedence effect offer potential explanations for these findings. We also found no significant difference between the β at all three velocities, suggesting that the physical audio-visual threshold in order to achieve a perceptual separation at the respective sensory peripheries is spatially invariant. Findings suggest a key role for auditory de-reverberation in processing moving auditory stimuli, informing the development of algorithms implemented in digital hearing aids, automatic speech recognition systems and telecommunications aimed at preserving speech intelligibility in reverberant spaces. The present result also establishes a perceptual measure for assessing the veracity of auditory motion generated from discrete spatial locations and in echoic environments.

## Supporting Information

Table S1 **PSE values for all 6 participants across the three acoustic conditions and three experimental velocities tested in experiments 1 and 2.**
(PDF)

## References

[1] Stein BE, Meredith MA (1993) The Merging of the Senses. Cambridge: The MIT Press.

[2] Wallace MT, Roberson GE, Hairston WD, Stein BE, Vaughan JW, et al (2004) Unifying multisensory signals across time and space. Exp Brain Res 158(2): 252–258. 15112119 10.1007/s00221-004-1899-9

[3] Meyer GF, Wuerger SM (2001) Cross-modal integration of auditory and visual motion signals. Neuroreport 12(11): 2557–2560. 11496148 10.1097/00001756-200108080-00053

[4] Schmiedchen K, Freigang C, Nitsche I, Rübsamen R (2012) Crossmodal interactions and multisensory integration in the perception of audio-visual motion — A free-field study. Brain Res 1466(C): 99–111. 22617375 10.1016/j.brainres.2012.05.015

[5] Alink A, Euler F, Galeano E, Krugliak A, Singer W, et al (2012) Auditory motion capturing ambiguous visual motion. Front Psychol 2: 391. 22232613 10.3389/fpsyg.2011.00391 PMC3249388

[6] Lewald J (2013) Exceptional ability of blind humans to hear sound motion Implications for the emergence of auditory space. Neuropsychologia 51(1): 181–186. 23178211 10.1016/j.neuropsychologia.2012.11.017

[7] Getzmann S, Lewald J, Guski R (2004) Representational momentum in spatial hearing. Perception 33(5): 591–599 DOI:10.1068/p5093 15250664 10.1068/p5093

[8] Carlile S (1996) Virtual auditory space: Generation and applications. Austin TX, USA: RG Landes.

[9] Chandler DW, Grantham DW (1992) Minimum audible movement angle in the horizontal plane as a function of stimulus frequency and bandwidth, source azimuth, and velocity. J Acoust Soc Am 91(3): 1624–1636. 1564199 10.1121/1.402443

[10] Perrott DR, Marlborough K (1989) Minimum audible movement angle: marking the end points of the path traveled by a moving sound source. J Acoust Soc Am 85(4): 1773–1775. 2708691 10.1121/1.397968

[11] Wuerger S, Meyer G, Hofbauer M, Zetzsche C, Schill K (2010) Motion extrapolation of auditory–visual targets. Inform Fusion 11(1): 45–50.

[12] Perrott DR, Musicant AD (1977) Minimum auditory movement angle: binaural localization of moving sound sources. J Acoust Soc Am 62(6): 1463–1466. 591679 10.1121/1.381675

[13] Grantham DW (1986) Detection and discrimination of simulated motion of auditory targets in the horizontal plane. J Acoust Soc Am 79(6): 1939–1949. 3722604 10.1121/1.393201

[14] Carlile S, Best V (2002) Discrimination of sound source velocity in human listeners. J Acoust Soc Am 111(2): 1026–35. 11863159 10.1121/1.1436067

[15] Freeman TC, Leung J, Wufong E, Orchard-Mills E, Carlile S, et al (2014) Discrimination Contours for Moving Sounds Reveal Duration and Distance Cues Dominate Auditory Speed Perception. PloS one 9(7): e102864. 25076211 10.1371/journal.pone.0102864 PMC4116163

[16] Hartmann WM (1983) Localization of sound in rooms. J Acoust Soc Am 74(5): 1380–1391. 6643850 10.1121/1.390163

[17] Giguere C, Abel S (1993) Sound localization: Effects of reverberation time, speaker array, stimulus frequency and stimulus rise/decay. J Acoust Soc Am 94: 769–776. 8370883 10.1121/1.408206

[18] Mershon DH, King LE (1975) Intensity and reverberation as factors in the auditory perception of egocentric distance. Percept Psychophys 18(6): 409–415.

[19] Zurek PM, Freyman RL, Balakrishnan U (2004) Auditory target detection in reverberation. J Acoust Soc Am 115: 1609–1620. 15101640 10.1121/1.1650333

[20] Zahorik P, Brungart DS, Bronkhorst AW (2005) Auditory distance perception in humans: A summary of past and present research. Acta Acust United Ac 91(3): 409–420.

[21] Shinn-Cunningham BG (2000) Distance cues for virtual auditory space. IEEE-PCM 2000, Sydney, Australia. 227–230.

[22] Albright TD, Stoner GR (1995) Visual motion perception. Proc Natl Acad Sci U.S.A. 92 (7): 2433–2440. 7708660 10.1073/pnas.92.7.2433 PMC42232

[23] Boucher L, Lee A, Cohen YE, Hughes HC (2004) Ocular tracking as a measure of auditory motion perception. J Physiol 98(1): 235–248. 10.1016/j.jphysparis.2004.03.010 15477035

[24] Grantham DW (1997) Auditory motion perception: Snapshots revisited. In: Gilkey R, Anderson T, editors. Binaural and spatial hearing in real and virtual environments. pp. 295–313.

[25] Saberi K, Petrosyan A (2006) Effects of interaural decorrelation and acoustic spectrum on detecting the motion of an auditory target. Acoust Phys 52(1): 87–92.

[26] Battaglia PW, Jacobs RA, Aslin RN (2003) Bayesian integration of visual and auditory signals for spatial localization. JOSA A 20(7): 1391–1397. 12868643 10.1364/josaa.20.001391

[27] Burr D, Alais D (2006) Combining visual and auditory information. Prog Brain Res 155: 243–258. 17027392 10.1016/S0079-6123(06)55014-9

[28] Ernst MO, Banks MS (2002) Humans integrate visual and haptic information in a statistically optimal fashion. Nature 415(6870): 429–433. 11807554 10.1038/415429a

[29] Alais D, Burr D (2004) The Ventriloquist Effect Results from Near-Optimal Bimodal Integration. Curr Biol 14(3): 257–262. 14761661 10.1016/j.cub.2004.01.029

[30] Hairston WD, Wallace MT, Vaughan JW, Stein BE, Norris JL, et al (2003) Visual localization ability influences cross-modal bias. J Cogn Neurosci 15(1): 20–29. 12590840 10.1162/089892903321107792

[31] Brooks A, Van Der Zwan R, Billard A, Petreska B, Clarke S, et al (2007) Auditory motion affects visual biological motion processing. Neuropsychologia 45: 523–530. 16504220 10.1016/j.neuropsychologia.2005.12.012

[32] Jain A, Sally SL, Papathomas TV (2008) Audiovisual short-term influences and aftereffects in motion: examination across three sets of directional pairings. J Vis 8(15): 7.1–7.13. 10.1167/8.15.7 19146291

[33] Sanabria D, Lupianez J, Spence C (2007) Auditory motion affects visual motion perception in a speeded discrimination task. Exp Brain Res 178: 415–421. 17372657 10.1007/s00221-007-0919-y

[34] Middlebrooks JC (1992) Narrow-band sound localization related to external ear acoustics. J Acoust Soc Am 92: 2607–2624. 1479124 10.1121/1.404400

[35] Farina A (2007) Advancements in impulse response measurements by sine sweeps. AES 122th Convention, Vienna, Austria.

[36] Griffiths TD, Warren JD (2004) What is an auditory object? Nat Rev Neurosci 5(11): 887–892. 15496866 10.1038/nrn1538

[37] Feinkohl A, Locke S, Leung J, Carlile S (2014) The effect of velocity on auditory representational momentum. JASA-EL 136: EL20 doi: 10.1121/1.4881318 10.1121/1.4881318 24993233

[38] Brainard DH (1997) The Psychophysics Toolbox. Spatial Vision 10: 433–436. 9176952

[39] Pelli DG (1997) The VideoToolbox software for visual psychophysics: Transforming numbers into movies. Spatial Vision 10: 437–442. 9176953

[40] Kleiner M, Brainard D, Pelli D, Ingling A, Murray R, et al (2007) What's new in Psychtoolbox-3. Perception 36(14): 1–1.

[41] Wichmann FA, Hill NJ (2001) The psychometric function: I. Fitting, sampling, and goodness of fit. Percept Psychophys 63(8): 1293–1313. 11800458 10.3758/bf03194544

[42] Wichmann FA, Hill NJ (2001) The psychometric function: II. Bootstrap-based confidence intervals and sampling. Percept Psychophys 63(8): 1314–1329. 11800459 10.3758/bf03194545

[43] Efron B, Hinkley DV (1978) Assessing the accuracy of the maximum likelihood estimator: Observed versus expected Fisher information. Biometrika 65(3): 457–483.

[44] Jeub M, Nelke C, Beaugeant C, Vary P (2011) Blind estimation of the coherent-to-diffuse energy ratio from noisy speech signals. Proceedings of 19^th^ European Signal Processing Conference (EUSIPCO 2011), Barcelona, Spain.

[45] Zahorik P (2002) Direct-to-reverberant energy ratio sensitivity. J Acoust Soc Am 112(5): 2110. 12430822 10.1121/1.1506692

[46] Clifton RK, Freyman RL, Meo J (2002) What the precedence effect tells us about room acoustics. Percept Psychophys 64(2): 180–188. 12013373 10.3758/bf03195784

[47] Litovsky RY, Colburn HS, Yost WA, Guzman SJ (1999) The precedence effect. J Acoust Soc Am 106(4): 1633–1654. 10530009 10.1121/1.427914

[48] Blauert J (1997) Spatial Hearing: The Psychophysics of Human Sound Localization, Revised Edition. Cambridge: The MIT Press.

[49] Shinn-Cunningham B (2013) Auditory Precedence Effect. doi:10.1007/978-1-4614-7320-6_101-5.

[50] Zurek PM (1980) The precedence effect and its possible role in the avoidance of interaural ambiguities. J Acoust Soc Am 67: 952–964. 10.1121/1.383974 7358920

