# Supplementary material for: Effects of Virtual Speaker Density and Room Reverberation on Spatiotemporal Thresholds of Audio-Visual Motion Coherence
Source: PLoS One. 2014 Sep 30;9(9):e108437. doi: 10.1371/journal.pone.0108437 (PMC4182487; doi:10.1371/journal.pone.0108437)
Supplement: Table S1 — PSE values for all 6 participants across the three acoustic conditions and three experimental velocities tested in experiments 1 and 2. (PDF) [file pone.0108437.s001.pdf]

Table S1: Individual PSE values for all 6 participants across the three acoustic conditions and three experimental velocities tested in experiments 1 and 2.

Stimulus Velocity	25°/s	50°/s	100°/s
Subject No.	Anechoic (1°)		
1	-0.61	1.45	0.00
2	1.69	0.99	-0.16
3	0.30	-1.74	-2.78
4	1.07	0.28	-0.49
5	2.27	1.02	3.60
6	2.53	2.54	-1.98
Subject No.	Anechoic (5°)		
1	0.24	0.84	1.04
2	1.85	0.43	-1.85
3	-0.86	-1.81	-4.33
4	-1.37	-2.98	-3.01
5	1.20	1.68	2.99
6	1.59	1.54	-1.56
Subject No.	Reverberant (5°)		
1	-0.61	1.90	0.24
2	0.66	0.33	0.24
3	-0.02	-1.82	-4.41
4	-2.21	-2.54	-3.01
5	1.20	0.86	3.55
6	1.48	1.91	-1.67
